# Keep the Sick Clean and Cool

**Published:** 1886-07

**Authors:** 


### KEEP THE SICK CLEAN AND COOL.

In almost all affections, the function of the skin is more or less disturbed ; and in many important diseases, nature relieves herself almost entirely through the skin. The poisonous materials are merely thrown out by the skin, not carried away from the body by it. Nothing but soap and water can do that. If we permit the sick to remain unwashed, or their clothing to be worn after it has become saturated with perspiration or other excretions, we interfere just as much with the natural process as if a slow poison were given by the mouth ; only it is not so rapid in its operation.

None but those who have been sick, and know from personal experience, can tell how much delicious comfort may be secured after the skin has been carefully washed and properly *dried.* It is not the mere *feeling* of comfort which has been obtained, but it is a sign that the vital powers have been relieved by removing something which was *oppressing* them.

Cleanliness of skin and ventilation have much the same end in view, the removal of noxious materials from the system as rapidly as possible. Physicians are more ready to advise bathing than they were a few years ago, yet many ignore the use of water as a hindrance to the effect of their medicines, but the position to be taken in such cases is, to tell the physician that "cleanliness is akin to godliness," and that it is a thing not to be neglected.

Care should be taken, in all operations of sponging, washing, and cleansing the skin, not to expose too great a surface of the body at once, so as to check the perspiration, which might retard recovery from the disease, or renew the trouble in some other form. In several varieties of diarrhœa, dysentery, etc., when the skin is hard and harsh, the relief to the sick person from washing with water and using a good deal of soap, is almost beyond calculation.

In other cases, sponging with tepid water and soap will be ordered, then tepid water alone, and afterward properly drying the skin with a soft warm towel. Sometimes when water alone is to be used, a little vinegar added to it makes the sponging more refreshing. Of course, no one would think of using vinegar at the same time soap is used. Bay rum is very acceptable, also, to the face, neck, and hands of sick people, when used after sponging or bathing. If not convenient, some common spirits diluted with water may be substituted.

In this connection, it may be well to remark that special care should be observed in the use of water for bathing, in persons suffering with debility, the result of sickness or of age. In such persons, it is often seen that a bath used with benefit in robust health, or when younger, under other circumstances is followed by palpitation of the heart, slackened pulse, more or less vertigo, shivering, and other feelings of discomfort, lasting some time after its use.

